# Long-term patient-reported outcomes following congenital heart surgery in adults

**DOI:** 10.3389/fcvm.2024.1501680

**Published:** 2024-12-11

**Authors:** Thibault Schaeffer, Pauline Bossers, Doris Kienmoser, Oktay Tutarel, Paul Philipp Heinisch, Masamichi Ono, Julie Cleuziou, Jelena Pabst von Ohain, Jürgen Hörer

**Affiliations:** ^1^ Department of Congenital and Pediatric Cardiac Surgery, German Heart Center Munich, Technical University of Munich, Munich, Germany; ^2^ Division of Congenital and Pediatric Cardiac Surgery, University Hospital of Munich, Ludwig-Maximilians University Munich, Munich, Germany; ^3^ International Center for Adults with Congenital Heart Disease, Department of Congenital Heart Disease and Pediatric Cardiology, German Heart Center Munich, Technical University of Munich, Munich, Germany; ^4^ Department of Congenital and Pediatric Cardiac Surgery, Klinikum Stuttgart, Stuttgart, Germany; ^5^ Technical University of Munich, Munich, Germany

**Keywords:** adult with congenital heart disease, cardiac surgery, patient-reported outcomes, quality of life, illness perception

## Abstract

**Objective:**

To investigate the long-term impact of cardiac surgery on the quality of life in adults with congenital heart disease (ACHDs).

**Methods:**

Patients who had undergone cardiac surgery for congenital heart disease (CHD) at the age of 18 years or more were recruited in a single-center, cross-sectional study. The enrolled subjects completed online questionnaires to assess patient-reported outcomes: perceived health status and life satisfaction, psychological functioning, health behaviors, and illness perception. Clinical variables were correlated to the score results, and results were compared to representative samples from international and German national surveys of healthy subjects and ACHDs.

**Results:**

We enrolled 196 ACHDs (54% women), including 55% with more than one cardiac surgery during their lifetime. The median age at the survey was 43 years, with a median of 13 years since their last cardiac surgery. The majority of patients reported improved subjective wellbeing after cardiac operation and were in functional New York Heart Association class I or II. The severity of underlying CHD, number of previous cardiac operations, and beta-blocker medication had the most substantial negative effects on illness perception. Measured quality of life and health risk behaviors were within the range of values internationally reported for healthy and CHD subjects, respectively.

**Conclusions:**

ACHD, several years after cardiac surgery, reported a subjective improvement in their wellbeing, a life satisfaction comparable to that of healthy individuals, and low health risk behaviors. Illness perception is strongly correlated with the severity of the underlying CHD.

## 1 Introduction

Advances in cardiac surgery over the last few decades have made it possible for over 90% of patients with congenital heart disease (CHD) to reach adulthood, with two-thirds of this population now being adults (1). Thus, the number of adults with congenital heart disease (ACHDs) is expected to increase in the coming years, raising more and more questions about their medical and surgical management. The heterogeneity of this population makes it complex to study. Indeed, ACHDs represent a spectrum of patients, ranging from chronically ill patients from birth, with cardiac and non-cardiac comorbidities and multiple cardiac interventions in their history, to asymptomatic patients whose congenital anomaly was incidentally revealed in adulthood. Regardless of their profile, ACHDs are likely to undergo primary or redo cardiac surgery, and the question arises as to the impact of this surgery on their quality of life as adults.

Quality of life (QoL) is a broad concept of individual wellbeing measured by patient-reported outcomes (PROs) (2). Alongside QoL, PROs enable the study of patients’ perceptions of health and illness, functional status, and psychological functioning. The “Assessment of Patterns of Patient-Reported Outcomes in Adults with Congenital Heart Disease—International Study” (APPROACH-IS) was designed to explore these issues in the ACHD population, using a uniform methodology that could be applied in various regions worldwide (3). The study has revealed geographical variations and provided new insights into the psychological, social, and behavioral factors influencing ACHDs’ QoL (4). However, no centers in Germany took part in this international project.

With the present study, based on elements of the APPROACH-IS methodology, we aimed to investigate the long-term impact of cardiac surgery in adulthood on QoL in patients with CHD from a German Center.

## 2 Material and methods

### 2.1 Study design

In this single-center, cross-sectional study, we selected patients with CHD who underwent cardiac surgery at the age of 18 years or older at the German Heart Center Munich between 2004 and 2013. For patients who underwent repeated operations in adulthood, the most recent operation was considered the reference point. Patients who had undergone isolated aortic valve procedures without prior cardiac intervention or operation before the age of 18 years were excluded from the patient selection. Furthermore, patients who had undergone cardiac transplantation, transvenous implantation of a pacemaker or implantable cardioverter-defibrillator (ICD) as an isolated procedure, and non-German-speaking patients were also excluded. Eligible subjects were invited to take part in the study. The recruited patients completed the surveys digitally via the Heartbeat Medical online platform (HRTBT Medical Solutions GmbH, Berlin, Germany). Patients’ clinical data were obtained after a review of the internal medical database.

### 2.2 Measured outcomes

The severity of CHD was defined according to the Bethesda classification (5). As not all were available in German, the surveys differed slightly from those of the APPROACH-IS (3, 6–12). The following surveys (German version) were selected for the assessment of the corresponding PROs:

1. Perceived health status and life satisfaction:
  a. Veterans RAND 12-item mental and physical scores (VR-12)
  b. EuroQol 5-Dimension Level (EQ-5D-5l)
  c. Satisfaction with Life Scale (SWLS)
2. Psychological functioning (depression and sense of coherence):
  a. Patient Health Questionnaire (PHQ-8)
  b. Sense of Coherence (SOC-13)
3. Health risk behaviors: Health-Behavior Scale—Congenital Heart Disease (HBS-CHD)
4. Illness perception: Illness Perception Questionnaire-Revised (IPQ-R)

Details of the questionnaires and their scope of investigation are provided in Supplementary Table S1. An additional institutional questionnaire asked the patients about their social and demographic situation, functional status [New York Heart Association (NYHA) score], and subjective improvement in their wellbeing since their last cardiac operation. All questionnaires submitted to patients (in German) are available in the Supplementary Material.

### 2.3 Statistical analysis

Continuous variables were reported as medians with interquartile ranges. Categorical variables were reported as frequencies with percentages. Score results were reported as medians with ranges (minimum–maximum) and mean with standard deviations. Demographic, medical, and surgical factors affecting a relevant percentage of subjects were selected as potential explanatory variables for the variation in score results. These included gender, severity of CHD, number of previous cardiac operations, age at first cardiac operation, age at last cardiac operation, age at study, presence of pacemaker or ICD, anticoagulant and beta-blocker therapy. As the scores of the scales in their original form have varying values and polarities, all scores were rescaled from 0 to 100 (0 being the worst subjective value and 100 being the best) to better illustrate their relationship with the clinical factors. The effect size was estimated using Pearson's correlation coefficient. *P*-values <0.05 were considered statistically significant.

To refine the granularity of the information reported by this study on ACHDs with different diagnoses and medical histories, we divided the patients into three groups according to the complexity of their primary diagnosis defined by the Bethesda classification: simple, moderate, or severe CHD. A comparison of patient characteristics and PROs between the groups was performed using a Kruskal–Wallis test or a pairwise Fisher's exact test as appropriate.

As absolute score values provide limited information, we compared the data obtained with those available in the literature on healthy subjects. However, the Health-Behavior Scale—Congenital Heart Disease was compared with young adults with CHD (median age 16.3 years), as the questionnaire was not administered to healthy subjects. After selecting studies with documented sample sizes, mean values, and standard deviations of the scores, we compared the results using Welch's *t*-test (11, 13–17). For the PHQ-8, a two-proportion Z-test was applied for patients reporting a score ≥10, which corresponds to the cut-off point for depression (16). Due to the mismatch between the questionnaires and the geographical diversity, we refrained from statistical comparisons with the APPROACH-IS and only reported its results for information. For better comprehension, the score results have also been rescaled from 0 to 100 for their graphical representation.

Statistical analysis and graphs were conducted and created with R, respectively (R Foundation for Statistical Computing, Vienna, Austria).

### 2.4 Ethical statement

This study was approved by the Institutional Review Board of the Technical University of Munich (No. 145/20 S-KH on 28/04/2020). All recruited patients consented to the anonymous processing and publication of their data.

## 3 Results

### 3.1 Demographic, clinical, and sociodemographic characteristics

After pre-selection, according to the criteria set out in the Methods section, 670 subjects were deemed eligible and invited to participate in the study. In total, 196 patients completed the questionnaires (participation rate of 29%). Patient characteristics with between-group comparisons are listed in Table 1. The median age at the time of the study was 43 years (37–53 years), and the median time since the last cardiac operation was 13.4 years (10.6–15.5 years). Approximately half of the patients had undergone more than one cardiac operation in their lifetime (55%). The severity of CHD was evenly distributed. Patients with severe CHD had a notably higher number of previous cardiac surgeries, were younger at the time of their first operation, and had a higher incidence of pacemaker or ICD implantation. Female patients were more represented in the group with simple CHD. The most frequent primary cardiac diagnoses were atrial septal defect (*n* = 34, 17%), tetralogy of Fallot (*n* = 22, 11%), and mixed aortic valve disease (*n* = 11, 6%). The most frequent cardiac procedures in adulthood were patch repair of an atrium septum defect (*n* = 31, 16%), replacement of a right ventricle-to-pulmonary artery conduit (*n* = 21, 11%), and mitral valvuloplasty (*n* = 20, 10%). Supplementary Tables S2, S3 provide complete lists of the patients’ primary cardiac diagnoses and cardiac procedures in adulthood, respectively. The analysis of the following data of the patients who were contacted but did not respond to the questionnaires (*n* = 474) revealed no statistically significant differences with the study sample: sex (male patients 47% vs. 46%, *P* = 0.9), age at last heart operation [30 (23–40) vs. 30 (24–41) years, *P* = 1], age at the time of the study [43 (36–53) vs. 43 (37–53) years, *P* = 0.9], and complexity of the congenital heart defect according to the Aristotle comprehensive complexity score [9.5 (7.0–11.5) vs. 9.5 (6.2–11.0) points, *P* = 1, the Bethesda score not being available for these patients].

**Table 1:** Patient characteristics.

	All (*n* = 196)	Mild CHD (*n* = 52)	Moderate CHD (*n* = 81)	Severe CHD (*n* = 63)	
	*N* (%)/median [IQR]				*P*-value
Demographics					
Female participants	106 (54%)	39 (75.0%)	38 (46.9%)	29 (46.0%)	**0**.**002**
Age at study (years)	43 [37–53]	42 [36–56]	43 [38–53]	43 [38–49]	0.4
Surgical characteristics					
Age at last cardiac surgery (years)	30 [24–40]	28 [20–42]	32 [25–40]	28 [23–35]	0.2
Time between study and last cardiac surgery (years)	13.4 [10.6–15.5]	13.3 [10.8–15.3]	12.6 [9.4–15.5]	14.0 [11.6–15.4]	0.2
Previous cardiac operation	108 (55%)	3 (5.8%)	43 (53.1%)	62 (98.4%)	**<0**.**001**
Number of previous cardiac operations					**<0**.**001**
1	48 (24%)	3 (5.8%)	22 (27.2%)	23 (36.5%)	
2	34 (17%)	0 (0.0%)	12 (14.8%)	22 (34.9%)	
3	17 (9%)	0 (0.0%)	8 (9.9%)	9 (14.3%)	
4	5 (3%)	0 (0.0%)	1 (1.2%)	4 (6.3%)	
5	3 (1.5%)	0 (0.0%)	0 (0.0%)	3 (4.8%)	
6	1 (0.5%)	0 (0.0%)	0 (0.0%)	1 (1.6%)	
Age at first cardiac surgery (years)	19.2 [2.4–30.8]	25.6 [19.9–41.6]	23.5 [7.4–33.7]	1.7 [0.2–4.9]	**<0**.**001**
Comorbidity					
Pacemaker and ICD	32 (16%)	1 (1.9%)	9 (11.1%)	22 (34.9%)	**<0**.**001**
Hypertension	31 (16%)	7 (13.5%)	17 (21.0%)	7 (11.1%)	0.2
Dyslipidemia	27 (14%)	6 (11.5%)	11 (13.6%)	10 (15.9%)	0.9
Diabetes mellitus	4 (2%)	2 (3.8%)	1 (1.2%)	1 (1.6%)	0.5
Obesity (BMI > 30 kg/m^2^)	23 (12%)	8 (15.4%)	8 (9.9%)	9 (14.3%)	0.3
Oncological disease	10 (5%)	4 (7.7%)	3 (3.7%)	3 (4.8%)	0.5
Thyroid disease substitution	25 (13%)	5 (9.6%)	11 (13.6%)	9 (14.3%)	0.8
Obstructive pulmonary disease	8 (4.1%)	1 (1.9%)	5 (6.2%)	2 (3.2%)	0.4
Neurological disease	25 (12.8%)	5 (9.6%)	7 (8.6%)	13 (20.6%)	0.11
Scoliosis	12 (6.1%)	1 (1.9%)	4 (4.9%)	7 (11.1%)	0.14
Intellectual disability	8 (4.1%)	0 (0.0%)	4 (4.9%)	4 (6.3%)	0.2
Medication					
Beta-blockers	76 (39%)	8 (15.4%)	32 (39.5%)	36 (57.1%)	**<0**.**001**
Anticoagulants	65 (33%)	5 (9.6%)	36 (44.4%)	24 (38.1%)	**<0**.**001**
Diuretics	30 (15%)	6 (11.5%)	9 (11.1%)	15 (23.8%)	0.12
Antipsychotics	9 (5%)	2 (3.8%)	3 (3.7%)	4 (6.3%)	0.8
Anti-epileptics	3 (2%)	0 (0.0%)	1 (1.2%)	2 (3.2%)	0.4

CHD, congenital heart disease; IQR, interquartile range; ICD, implantable cardiac defibrillator.

Values in bold indicate statistical significance.

Details of sociodemographic characteristics, as reported by the patients, are listed in Table 2. The majority of patients were married or in a partnership (70%), were engaged in a professional activity (85% with full-time or part-time jobs), and had a driver's license (94%). The patients in the group with simple CHD were more likely to be married or in a partnership and had more children than the other two groups.

**Table 2:** Sociodemographic characteristics.

	All (*n* = 196)	Mild CHD (*n* = 52)	Moderate CHD (*n* = 81)	Severe CHD (*n* = 63)	
	*N* (%)/median [IQR]				*P*-value
Married/partnership	137 (69.9%)	43 (82.7%)	54 (66.7%)	40 (63.5%)	0.059
Children	109 (55.6%)	38 (73.1%)	44 (54.3%)	27 (42.9%)	**0**.**005**
Education					0.7
University degree	64 (32.7%)	22 (42.3%)	23 (28.4%)	19 (30.2%)	
Training	115 (58.7%)	27 (51.9%)	49 (60.5%)	39 (61.9%)	
No training after graduation	11 (5.6%)	2 (3.8%)	6 (7.4%)	3 (4.8%)	
No school graduation	6 (3.1%)	1 (1.9%)	3 (3.7%)	2 (3.2%)	
Employment status					0.07
Full-time	64 (32.7%)	21 (40.4%)	24 (29.6%)	19 (30.2%)	
Part-time	102 (52.0%)	19 (36.5%)	45 (55.6%)	38 (60.3%)	
Job seeker	3 (1.5%)	2 (3.8%)	1 (1.2%)	0 (0.0%)	
Homemaker	5 (2.6%)	3 (5.8%)	0 (0.0%)	2 (3.2%)	
Retired	21 (10.7%)	7 (13.5%)	11 (13.6%)	3 (4.8%)	
Disability pension	1 (0.5%)	0 (0.0%)	0 (0.0%)	1 (1.6%)	
Driver’s license	185 (94.4%)	51 (98.1%)	76 (93.8%)	58 (92.1%)	0.4

CHD, congenital heart disease.

Values in bold indicate statistical significance.

### 3.2 Patient-reported outcomes

**Figure 1:**
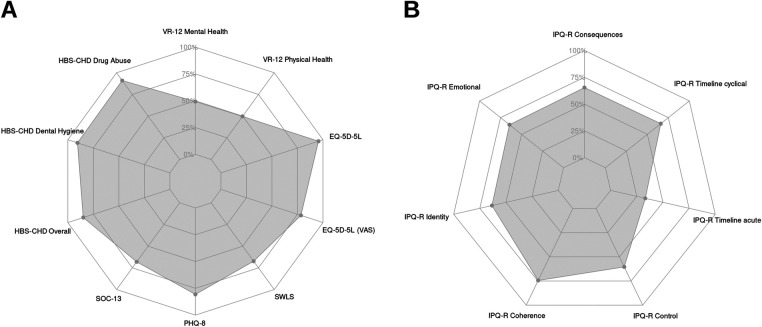
Radar chart of the patient-reported outcomes, rescaled from 0 to 100 (0 being the worst subjective value and 100 being the best). **(A)** Perceived health status and life satisfaction, psychological functioning, and health risk behaviors; **(B)** illness perception. VR-12, Veterans RAND 12-item mental and physical scores; EQ-5D-5l, EuroQol 5-Dimension Level; VAS, visual analog scale; SWLS, Satisfaction with Life Scale; PHQ-8, Patient Health Questionnaire; SOC-13, Sense of Coherence 13; HBS-CHD, Health-Behavior Scale—Congenital Heart Disease; IPQ-R, Illness Perception Questionnaire-Revised; CHD, congenital heart disease.

Score results are detailed in Table 3 and plotted in Figure 1. According to the institutional questionnaire, most patients reported improved wellbeing after the cardiac operation and were in functional NYHA class I or II. Except for the EQ-5D-5l (visual analog scale, VAS), which was slightly lower for the severe CHD group, the results regarding the patients’ perceived health status and life satisfaction, psychological functioning, and health risk behaviors were similar between the patient groups. Illness perception showed the most significant variation between the groups, with patients with severe CHD reporting significantly inferior results in all items except “Personal Control.”

**Table 3:** Patient-reported outcomes.

Outcome	All (*n* = 196)	Mild CHD (*n* = 52)	Moderate CHD (*n* = 81)	Severe CHD (*n* = 63)	*P*-value
	*N* (%)/median [range]				
Institutional questionnaire					
Overall wellbeing following cardiac surgery					0.2
Significantly improved	128 (65.3%)	32 (61.5%)	54 (66.7%)	42 (66.7%)	
Slightly improved	32 (16.3%)	8 (15.4%)	11 (13.6%)	13 (20.6%)	
Unchanged	29 (14.8%)	12 (23.1%)	11 (13.6%)	6 (9.5%)	
Worsened	7 (3.6%)	0 (0.0%)	5 (6.2%)	2 (3.2%)	
Functional class (NYHA)					0.08
I	123 (62.8%)	35 (67.3%)	56 (69.1%)	32 (50.8%)	
II	56 (28.6%)	15 (28.8%)	16 (19.8%)	25 (39.7%)	
III	16 (8.2%)	2 (3.8%)	9 (11.1%)	5 (7.9%)	
IV	1 (0.5%)	0 (0.0%)	0 (0.0%)	1 (1.6%)	
Perceived health status and life satisfaction					
VR-12 mental health	51.7 [16.7–65.2]	52.5 [16.7–65.2]	51.6 [22.6–63.3]	50.0 [20.8–64.7]	0.5
VR-12 physical health	52 [27–64]	54 [32–62]	53 [28–62]	48 [27–64]	0.09
EQ-5D-5l	98.0 [50–100]	95 [50–100]	98 [79–100]	98 [68–100]	0.6
EQ-5D-5l (VAS)	82 [20–100]	85 [20–100]	81 [24–100]	80 [25–100]	**0**.**037**
SWLS	73.3 [0.0–100]	76.7 [0.0–100]	70.0 [10.0–100]	70.0 [3.3–96.7]	0.3
Psychological functioning					
PHQ-8	83.3 [12.5–100]	87.5 [12.5–100]	83.3 [33.3–100]	79.2 [33.3–100]	0.11
SOC-13	70 [26–100]	72 [29–99]	71 [27–96]	64 [26–100]	0.2
Health risk behaviors					
HBS-CHD overall	86 [29–100]	86 [57–100]	86 [29–100]	86 [43–100]	0.5
HBS-CHD dental hygiene	100 [33–100]	100 [33–100]	100 [33–100]	100 [33–100]	0.9
0	6 (3.1%)	1 (1.9%)	2 (2.5%)	3 (4.8%)	
33	44 (22.4%)	12 (23.1%)	18 (22.2%)	14 (22.2%)	
67	146 (74.5%)	39 (75.0%)	61 (75.3%)	46 (73.0%)	
HBS-CHD substance use	0 [0–100]				0.3
0	2 (1.0%)	0 (0.0%)	2 (2.5%)	0 (0.0%)	
33	7 (3.6%)	2 (3.8%)	2 (2.5%)	3 (4.8%)	
67	33 (16.8%)	6 (11.5%)	18 (22.2%)	9 (14.3%)	
100	154 (78.6%)	44 (84.6%)	59 (72.8%)	51 (81.0%)	
Illness perception (IPQ-R)					
Consequences	75.0 [0.0–100]	87.5 [0.0–100]	75.0 [0.0–100]	57.5 [0.0–100]	**<0**.**001**
Emotional representations	67.5 [7.5–100]	75.0 [7.5–100]	67.5 [17.5–100]	57.5 [7.5–100]	**0**.**002**
Identity	64.3 [3.6–100]	71.4 [25.0–100]	67.9 [7.1–100]	53.6 [3.6–100]	**<0**.**001**
Coherence	75.0 [0.0–100]	82.5 [17.5–100]	75.0 [0.0–100]	75.0 [17.5–100]	**0**.**043**
Personal control	67.5 [0.0–100]	57.5 [17.5–100]	67.5 [0.0–100]	57.5 [0.0–100]	0.15
Acute/chronic timeline	25.0 [0.0–100]	71.3 [0.0–100]	25.0 [0.0–100]	0.0 [0.0–57.5]	**<0**.**001**
Cyclical timeline	67.5 [0.0–100]	82.5 [0.0–100]	67.5 [17.5–100]	57.5 [17.5–100]	**0**.**005**

NYHA, New York Heart Association; VR-12, Veterans RAND 12-item mental and physical scores; EQ-5D-5l, EuroQol 5-Dimension Level; VAS, visual analog scale; SWLS, Satisfaction with Life Scale; PHQ-8, Patient Health Questionnaire; SOC-13, Sense of Coherence 13; HBS-CHD, Health-Behavior Scale—Congenital Heart Disease; IPQ-R, Illness Perception Questionnaire-Revised.

Values in bold indicate statistical significance.

### 3.3 Correlation of scores with clinical factors

**Figure 2:**
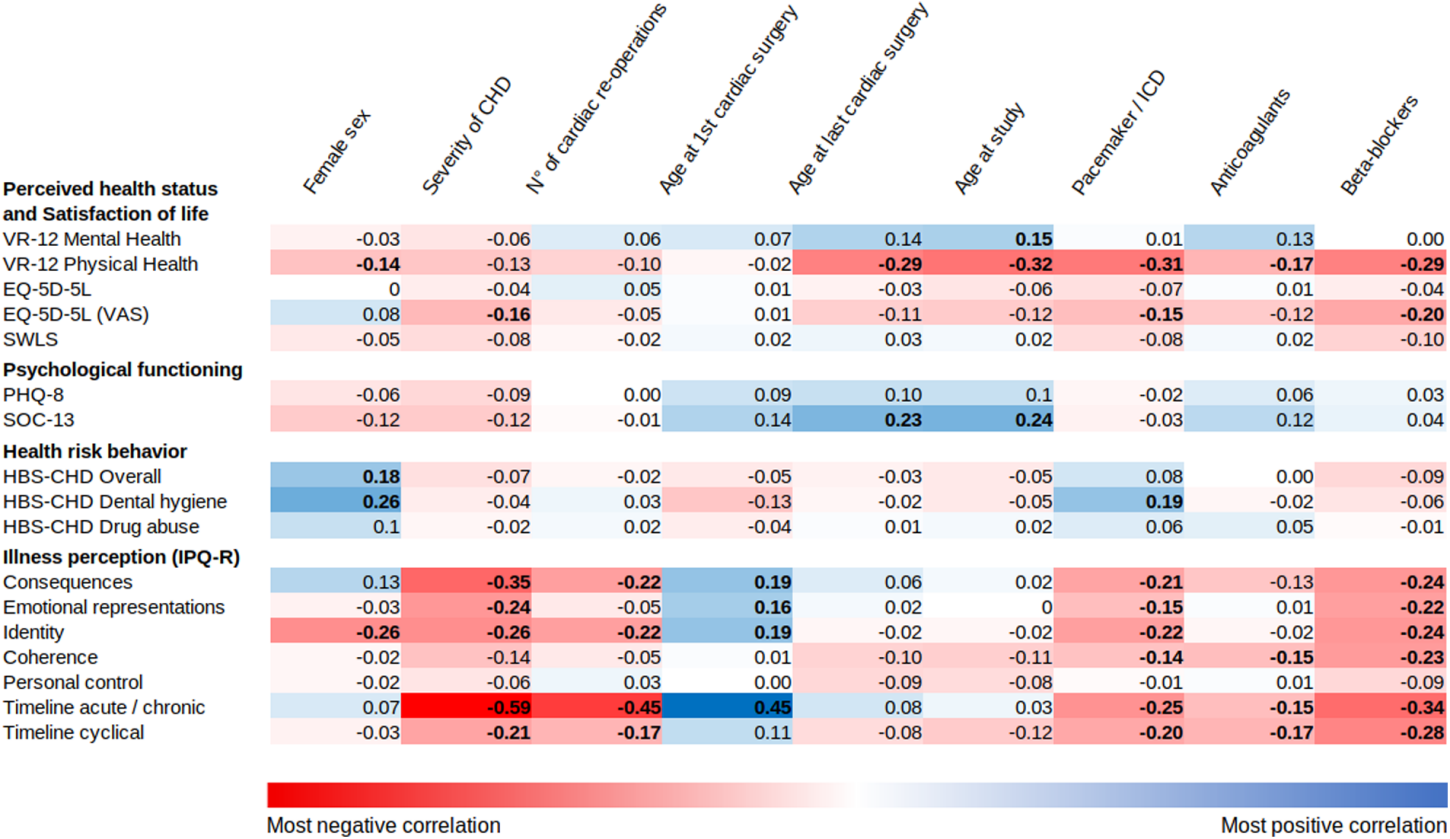
Correlations between clinical factors and the patient-reported outcomes rescaled from 0 to 100. The coefficients (*r*) from Pearson's correlation test are displayed. Positive correlations are highlighted in blue and negative correlations in red. Bold values indicate a significant *P*-value.

The results of the correlation analysis are depicted in Figure 2. Older age at last cardiac surgery, older age during the study, having a pacemaker, and beta-blocker medication were all associated with worse perceived physical health. Aging was associated with better perceived mental health and greater coherence. Female patients reported significantly lower risks in overall health behavior and dental hygiene.

Of all the factors, the PROs from the IPQ-R showed the most remarkable occurrence of statistically significant associations with the investigated variables, especially the “Consequences” (i.e., the believed impact of the disease on various aspects of the patient's life), “Timeline acute/chronic” (i.e., the perceived duration of the disease), and “Identity” (i.e., the symptoms the patient attributes to the illness) items. The severity of CHD, number of previous cardiac operations, and beta-blocker medication had the strongest substantial negative correlations with illness perception. In contrast, older age at first cardiac surgery had the most significant positive correlation.

### 3.4 Comparison with representative samples

**Figure 3:**
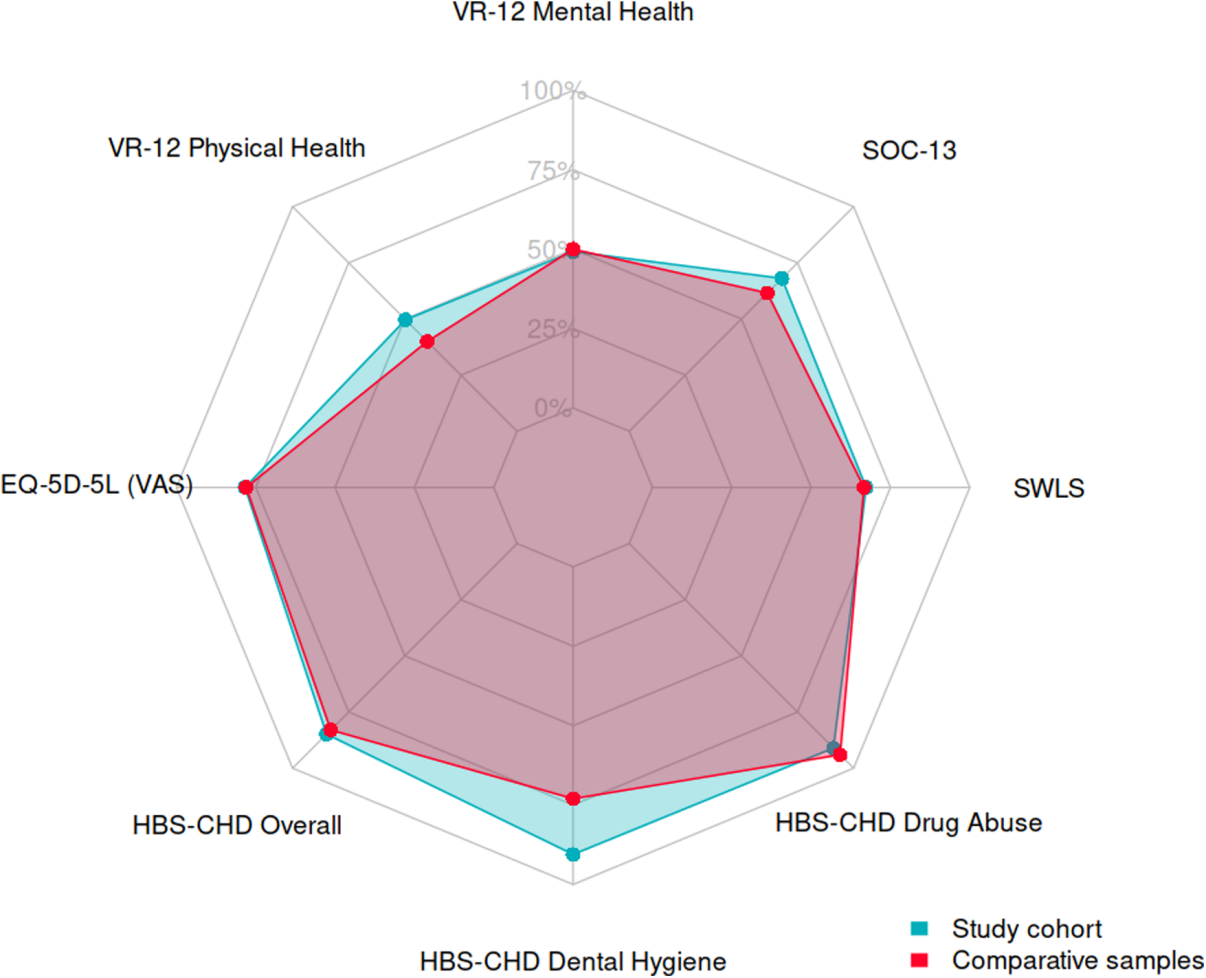
Radar chart comparing the patient-reported outcomes between the study cohort (blue) and selected samples (red), with scores rescaled from 0 to 100. VR-12, Veterans RAND 12-item mental and physical scores; EQ-5D-5l, EuroQol 5-Dimension Level; SWLS, Satisfaction with Life Scale; PHQ-8, Patient Health Questionnaire, SOC-13, Sense of Coherence 13; HBS-CHD, Health-Behavior Scale—Congenital Heart Disease.

Details of the comparison of patient-reported outcomes with representative samples are listed in Table 4, and the results are plotted in Figure 3. The study population presented significantly better physical health perceptions and a sense of coherence than the available data on healthy subjects. Furthermore, the data showed a lower risk of dental hygiene compared with the selected sample of young adults with CHD. The results reported by APPROACH-IS for scores equivalent to our study are also listed in Table 4 as a reference.

**Table 4:** Comparison of patient-reported outcomes with representative samples.

	Study cohort (*n* = 196)	Comparative samples					*P-*value	APPROACH-IS (*n* = 4,028)
	Mean (SD)/%	Reference	Year	Country	*n*	Mean (SD)/%		Mean (SD)/%
Healthy subjects								
Perceived health status and life satisfaction								
VR-12 mental health	49.2 (10.3)	Selim et al. (13)	2009	USA	52,425	50.0 (11.49)	0.3	
VR-12 physical health	49.7 (7.9)	*id*	*id*	*id*	*id*	39.8 (12.28)	**<0.001**	
EQ-5D-5l (VAS)	79.0 (17)	Ludwig et al. (14)	2018	Germany	1,158	78.0 (17)	0.7	77.9 (16.5)
SWLS	25.2 (6.3)	Glaesmer et al. (15)	2011	Germany	2,519	25.0 (6)	0.7	25.4 (6.6)
Psychological functioning								
PHQ-8	4.6 (3.9)							3.2 (3.3)
PHQ-8 ≥ 10	12.2%	Bretschneider et al. (16)	2017	Germany	23,602	10.1%	0.4	
SOC-13	66.1 (13.4)	Pallant and Lae (17)	2002	Germany	422	61.0 (12)	**<0.001**	65.5 (13.2)
Patients with CHD								
Health risk behaviors								
HBS-CHD overall	15.1 (14.5)	Goossens et al. (11)	2013	Belgium	401	17.0 (14)	0.2	22.6 (17.1)
HBS-CHD dental hygiene	9.5 (17.1)	*id*	*id*	*id*	*id*	27.0 (23)	**<0.001**	
HBS-CHD substance use	9.0 (19.2)	*id*	*id*	*id*	*id*	6.0 (18)	0.1	

APPROACH-IS, Assessment of Patterns of Patient-Reported Outcomes in Adults with Congenital Heart Disease—International Study; SD, standard deviation; VR-12, Veterans RAND 12-item mental and physical scores; *id*, idem; EQ-5D-5l, EuroQol 5-Dimension Level; SWLS, Satisfaction with Life Scale; PHQ-8, Patient Health Questionnaire; SOC-13, Sense of Coherence 13; HBS-CHD, Health-Behavior Scale—Congenital Heart Disease.

*P*-values refer to statistical comparisons between the study population (German Heart Center Munich) and the comparative samples. Values in bold indicate statistical significance. Data from the APPROACH-IS are reported for information only.

## 4 Discussion

In this study, we aimed to assess the long-term QoL of patients with CHD after cardiac surgery in adulthood. Upon direct inquiry, the majority of patients reported a subjective improvement in their wellbeing after the cardiac operation. Perceived health status, life satisfaction, and psychological functioning were comparable to those of healthy subjects from Germany and other countries, as were the health risk behaviors compared to a representative sample of young adults with CHD. Of all the measured outcomes, illness perception was the most influenced by the investigated clinical factors, predominantly by the severity of CHD.

With more than 4,000 patients recruited from 15 different countries, the APPROACH-IS has provided rich information on the subjective wellbeing of ACHDs and their correlates (4). Medical, demographic, behavioral, psychological, and social factors were identified. Social factors appeared to be notably complex, involving individual (e.g., partnership, educational level, and employment status) and contextual elements (e.g., healthcare system resources and performance). Moreover, the study showed that the relative contribution of each factor varies between countries and cultures (18). Considering the results reported by the APPROACH-IS, QoL in our study cohort was within the international range. More importantly, the QoL of our patients several years after cardiac surgery was also equivalent to that of healthy subjects. This information must be considered when advising ACHDs who are undergoing primary or redo surgery and should also be familiar to their attending cardiologists. As a surgeon, one is often confronted with the reluctance of patients and/or their attending cardiologist to proceed with surgery when it is formally indicated. One of the reasons for this reluctance is the fear of loss of quality of life associated with cardiac surgery. However, postponing surgery is detrimental to cardiac function and may lead to higher morbidity and mortality (19). Our study showed that a population of CHD patients who underwent cardiac surgery in adulthood reported a good quality of life and subjective improvement of wellbeing following the operation. These observations should reassure the patients concerned and their attending cardiologist, limiting postponed operations and their harmful consequences.

Nevertheless, the shortcomings of these estimates and their limited discriminatory power should be noted. The results from the illness perception questionnaire remind us of the impact of the disease on the daily life of these patients, which may be insufficiently captured by QoL questionnaires. In line with other studies, we found that the severity of CHD is a crucial determinant of illness perception: the more severe the disease, the worse the illness perception (20, 21). The significant correlations observed between outcomes and disease severity suggest that the underlying disease confounds clinical factors such as the number of cardiac reoperations and cardiac comorbidities, which are more prevalent in patients with severe CHD. Moreover, the resilience—in this case, the ability to accept an impaired health status and adapt positively—of patients born with a congenital disease is a well-known phenomenon, whose influence on psychometric tests makes a comparison of the outcomes with healthy subjects problematic. As an illustration, Moons P. et al. demonstrated that a sense of coherence, a representative measure of resilience, positively influenced QoL in ACHDs, with substantial inter-country variation (22).

A methodological advantage of the APPROACH-IS protocol is that it covers a wide range of PROs whose items are conceptually clearly defined, and enables comparisons to be made thanks to its uniform nature. Nevertheless, not all aspects are covered by this protocol. In our study of patients who had undergone cardiac surgery in previous years, no questionnaire specifically addressed the issue of pain and functional status in the region of the sternotomy (or of any peripheral cannulation) or cosmetic considerations regarding the appearance of the scar. Similarly, social aspects were poorly investigated, and we have created an institutional questionnaire to compensate for this lack of information. Economic status, the influence of which on the quality of life in ACHDs has been documented by other authors, was also not analyzed (23). Finally, this protocol, like all questionnaires investigating patient subjectivity, is not free from measurement bias. We have already mentioned resilience in the chronically ill; inter-sex variations in the impact of symptoms on daily life, cognitive status, and any psychological disorders are other parameters with little or no measurability that may also have affected the results (24).

In any case, the generally good QoL in our cohort should not eclipse the occasional poor outcomes that reflect the distress experienced by the most severely ill patients. These cases should be acknowledged and, if necessary, addressed with appropriate diagnostic and therapeutic interventions. Specific studies of the most severely ill patients, such as those with univentricular hearts, have revealed lower QoL and more negative illness perceptions (21). To address this issue, we are conducting a study on the QoL of adult patients with Fontan circulation who were operated on at our center (25).

This study was limited by its single-center design, the number of participants, and their heterogeneous diagnoses and interventions. The samples selected for the comparative analysis are also heterogeneous and limited to the data available in the literature. Nevertheless, our aim was not to draw broadly applicable conclusions comparing ACHDs to healthy individuals, but to provide insights specific to an under-researched German cohort focusing on surgical outcomes.

## 5 Conclusions

Several years after cardiac surgery in adulthood, adults with congenital heart disease reported a subjective improvement in their wellbeing, a life satisfaction comparable to that of healthy individuals, and low health risk behaviors. Illness perception strongly correlated with the severity of the underlying congenital heart disease.

## Data Availability

The raw data supporting the conclusions of this article will be made available by the authors, without undue reservation.
