# Supplementary material for: Long-term patient-reported outcomes following congenital heart surgery in adults
Source: Front Cardiovasc Med. 2024 Dec 11;11:1501680. doi: 10.3389/fcvm.2024.1501680 (PMC11668799; doi:10.3389/fcvm.2024.1501680)
Supplement: Supplementary file 1 [file Datasheet1.pdf]

Fragebogen zum Allgemeinen Gesundheitszustand VR-12

In diesem Fragebogen geht es um die Beurteilung Ihres Gesundheitszustandes. Ihre Angaben ermöglichen es nachzuvollziehen, wie Sie sich fühlen und wie gut Sie Ihre Alltagstätigkeiten ausüben können.

Bitte beantworten Sie jede Frage, indem Sie die Antwortmöglichkeit ankreuzen, die am besten auf Sie zutrifft. Wenn Sie sich bei der Antwort auf eine Frage unsicher sind, beantworten Sie diese bitte so gut wie möglich.

	**aus-gezeichnet**	**sehr gut**	**gut**	**weniger gut**	**schlecht**
**1.** Wie würden Sie Ihren Gesundheitszustand im Allgemeinen beschreiben?	☐	☐	☐	☐	☐

In den folgenden Fragen geht es um Tätigkeiten, die Sie vielleicht im Laufe eines normalen Tages ausüben. Sind Sie derzeit aufgrund Ihrer Gesundheit bei diesen Tätigkeiten eingeschränkt? Wenn ja, wie stark?

	**ja, stark eingeschränkt**	**ja, etwas eingeschränkt**	**nein, überhaupt nicht eingeschränkt**
**2.** **mittelschwere Tätigkeiten**, z.B. einen Tisch verschieben, staubsaugen, kegeln....................................	☐	☐	☐
**3.** **mehrere** Treppenabsätze steigen.....................................	☐	☐	☐

Hatten Sie in den vergangenen 4 Wochen aufgrund Ihrer **körperlichen** Gesundheit die folgenden Probleme bei der Arbeit oder bei anderen Alltagstätigkeiten?

	**nie**	**selten**	**manchmal**	**meistens**	**immer**
**4.** Ich habe weniger geschafft als ich wollte	☐	☐	☐	☐	☐
**5.** Ich konnte nur bestimmte Arbeiten oder andere Tätigkeiten ausführen..	☐	☐	☐	☐	☐

Hatten Sie in den vergangenen 4 Wochen aufgrund **seelischer** Probleme die folgenden Schwierigkeiten bei der Arbeit oder bei anderen Alltagstätigkeiten (z.B. weil Sie sich niedergeschlagen oder ängstlich fühlten)?

	**nie**	**selten**	**manchmal**	**meistens**	**immer**
**6.** Ich habe weniger geschafft als ich wollte	☐	☐	☐	☐	☐
**7.** Ich konnte meine Arbeit oder andere Tätigkeiten nicht so sorgfältig wie sonst erledigen..................................	☐	☐	☐	☐	☐

	**überhaupt nicht**	**ein wenig**	**mäßig**	**ziemlich**	**sehr**
**8.** Inwieweit haben Schmerzen in den vergangenen 4 Wochen Ihre Alltagstätigkeiten (im Beruf und zu Hause) beeinträchtigt?	☐	☐	☐	☐	☐

In diesen Fragen geht es darum, wie Sie sich in den vergangenen 4 Wochen gefühlt haben und wie es Ihnen ergangen ist. Bitte kreuzen Sie bei jeder Frage die Antwort an, die am besten beschreibt, wie Sie sich gefühlt haben.

	**immer**	**meistens**	**ziemlich oft**	**manchmal**	**selten**	**nie**
Wie oft fühlten Sie sich in den vergangenen 4 Wochen…						
**9.** ruhig und gelassen?	☐	☐	☐	☐	☐	☐
**10.** voller Energie?..	☐	☐	☐	☐	☐	☐
**11.** entmutigt und traurig?...	☐	☐	☐	☐	☐	☐

	**immer**	**meistens**	**manchmal**	**selten**	**nie**
**12.** Wie häufig haben Ihre körperliche Gesundheit oder seelischen Probleme in den vergangenen 4 Wochen Ihre Kontakte zu anderen Menschen (Besuche bei Freunden, Verwandten usw.) beeinträchtigt?	☐	☐	☐	☐	☐

Veterans RAND 12 Gesundheitsfragebogen ("VR-12: How to create VR-12 scales and PCS/MCS summaries" © 2014 by Trustees of Boston University, Dt. Version: Buchholz & Kohlmann 2015

EuroQol Group EQ-5D-5L

Bitte kreuzen Sie unter jeder Überschrift DAS Kästchen an, das Ihre Gesundheit HEUTE am besten beschreibt.

BEWEGLICHKEIT / MOBILITÄT

Ich habe keine Probleme herumzugehen ❑
Ich habe leichte Probleme herumzugehen ❑
Ich habe mäßige Probleme herumzugehen ❑
Ich habe große Probleme herumzugehen ❑
Ich bin nicht in der Lage herumzugehen ❑

FÜR SICH SELBST SORGEN

Ich habe keine Probleme, mich selbst zu waschen oder anzuziehen ❑
Ich habe leichte Probleme, mich selbst zu waschen oder anzuziehen ❑
Ich habe mäßige Probleme, mich selbst zu waschen oder anzuziehen ❑
Ich habe große Probleme, mich selbst zu waschen oder anzuziehen ❑
Ich bin nicht in der Lage, mich selbst zu waschen oder anzuziehen ❑

ALLTÄGLICHE TÄTIGKEITEN *(z.B. Arbeit, Studium, Hausarbeit, Familien- oder Freizeitaktivitäten)*

Ich habe keine Probleme, meinen alltäglichen Tätigkeiten nachzugehen ❑
Ich habe leichte Probleme, meinen alltäglichen Tätigkeiten nachzugehen ❑
Ich habe mäßige Probleme, meinen alltäglichen Tätigkeiten nachzugehen ❑
Ich habe große Probleme, meinen alltäglichen Tätigkeiten nachzugehen ❑
Ich bin nicht in der Lage, meinen alltäglichen Tätigkeiten nachzugehen ❑

SCHMERZEN / KÖRPERLICHE BESCHWERDEN

Ich habe keine Schmerzen oder Beschwerden ❑
Ich habe leichte Schmerzen oder Beschwerden ❑
Ich habe mäßige Schmerzen oder Beschwerden ❑
Ich habe starke Schmerzen oder Beschwerden ❑
Ich habe extreme Schmerzen oder Beschwerden ❑

ANGST / NIEDERGESCHLAGENHEIT

Ich bin nicht ängstlich oder deprimiert ❑
Ich bin ein wenig ängstlich oder deprimiert ❑
Ich bin mäßig ängstlich oder deprimiert ❑
Ich bin sehr ängstlich oder deprimiert ❑
Ich bin extrem ängstlich oder deprimiert ❑

Germany (German) © 2009 EuroQol Group EQ-5D™ is a trade mark of the EuroQol Group

- Wir wollen herausfinden, wie gut oder schlecht Ihre Gesundheit HEUTE ist.
- Diese Skala ist mit Zahlen von 0 bis 100 versehen.
- 100 ist die <u>beste</u> Gesundheit, die Sie sich vorstellen können.
  0 (Null) ist die <u>schlechteste</u> Gesundheit, die Sie sich vorstellen können.
- Bitte kreuzen Sie den Punkt auf der Skala an, der Ihre Gesundheit HEUTE am besten beschreibt.
- Jetzt tragen Sie bitte die Zahl, die Sie auf der Skala angekreuzt haben, in das Kästchen unten ein.

IHRE GESUNDHEIT HEUTE =

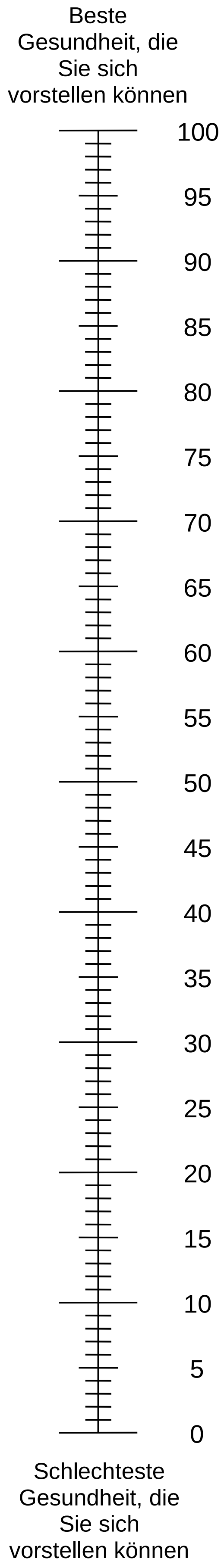

Germany (German) © 2009 EuroQol Group EQ-5D™ is a trade mark of the EuroQol Group

Deutsche Version der Satisfaction with Life Scale (SWLS)

Es folgen fünf Aussagen, denen Sie zustimmen bzw. die Sie ablehnen können. Bitte benutzen Sie die folgende Skala von 1-7, um Ihre Zustimmung bzw. Ablehnung zu jeder Aussage zum Ausdruck zu bringen.

	trifft überhaupt nicht zu	trifft nicht zu	trifft eher nicht zu	teils/ teils	trifft eher zu	trifft zu	trifft vollständig zu
In den meisten Bereichen entspricht mein Leben meinen Idealvorstellungen.	O	O	O	O	O	O	O
Meine Lebensbedingungen sind ausgezeichnet.	O	O	O	O	O	O	O
Ich bin mit meinem Leben zufrieden.	O	O	O	O	O	O	O
Bisher habe ich die wesentlichen Dinge erreicht, die ich mir für mein Leben wünsche.	O	O	O	O	O	O	O
Wenn ich mein Leben noch einmal leben könnte, würde ich kaum etwas ändern.	O	O	O	O	O	O	O

Gesundheitsfragebogen für Patienten (PHQ-8)

**Wie oft fühlten Sie sich im Verlauf der letzten 2 Wochen durch die folgenden Beschwerden beeinträchtigt?**	**Überhaupt nicht**	**An einzelnen Tagen**	**An mehr als der Hälfte der Tage**	**Beinahe jeden Tag**
Wenig Interesse oder Freude an Ihren Tätigkeiten	☐ 0	☐ 1	☐ 2	☐ 3
Niedergeschlagenheit, Schwermut oder Hoffnungslosigkeit.	☐ 0	☐ 1	☐ 2	☐ 3
Schwierigkeiten ein- oder durchzuschlafen oder vermehrter Schlaf	☐ 0	☐ 1	☐ 2	☐ 3
Müdigkeit oder Gefühl, keine Energie zu haben	☐ 0	☐ 1	☐ 2	☐ 3
Verminderter Appetit oder übermäßiges Bedürfnis zu essen	☐ 0	☐ 1	☐ 2	☐ 3
Schlechte Meinung von sich selbst; Gefühl, ein Versager zu sein oder die Familie enttäuscht zu haben	☐ 0	☐ 1	☐ 2	☐ 3
Schwierigkeiten, sich auf etwas zu konzentrieren, z.B. beim Zeitunglesen oder Fernsehen	☐ 0	☐ 1	☐ 2	☐ 3
Waren Ihre Bewegungen oder Ihre Sprache so verlangsamt, dass es auch anderen auffallen würde? Oder waren Sie im Gegenteil „zappelig“ oder ruhelos und hatten dadurch einen stärkeren Bewegungsdrang als sonst?	☐ 0	☐ 1	☐ 2	☐ 3

Gesamtwert _____ = Addition ____ + ____ + ____
der Spaltensummen

SOC-13

Im folgenden finden Sie eine Reihe von Fragen und Aussagen zu verschiedenen Aspekten des Lebens. Bitte **kreuzen** Sie zu jeder Frage die für Sie **persönlich zutreffende Antwort** (Zahl zwischen ① und ⑦) an. Bitte beantworten Sie **jede** Frage bzw. Aussage und kreuzen Sie **jeweils nur eine** Zahl an.

---

1. Haben Sie das Gefühl, dass es Ihnen ziemlich gleichgültig ist, was um Sie herum passiert?

① ② ③ ④ ⑤ ⑥ ⑦

sehr selten oder nie — sehr oft

---

2. Ist es in der Vergangenheit vorgekommen, dass Sie vom Verhalten von Menschen überrascht waren, die Sie gut zu kennen glaubten?

① ② ③ ④ ⑤ ⑥ ⑦

das ist nie passiert — das ist immer wieder passiert

---

3. Ist es vorgekommen, dass Sie von Menschen enttäuscht wurden, auf die Sie gezählt hatten?

① ② ③ ④ ⑤ ⑥ ⑦

das ist nie passiert — das ist immer wieder passiert

---

4. Bis jetzt hatte Ihr Leben...

① ② ③ ④ ⑤ ⑥ ⑦

überhaupt keine klaren Ziele — sehr klare Ziele

---

5. Haben Sie das Gefühl, dass Sie ungerecht behandelt werden?

① ② ③ ④ ⑤ ⑥ ⑦

sehr oft — selten oder nie

---

6. Haben Sie das Gefühl, dass Sie in einer ungewohnten Situation sind und nicht wissen, was Sie tun sollen?

① ② ③ ④ ⑤ ⑥ ⑦

sehr oft — sehr selten oder nie

---

7. Die Dinge, die Sie täglich tun, sind für Sie ...

① ② ③ ④ ⑤ ⑥ ⑦

eine Quelle tiefer Freude und Befriedigung. — eine Quelle von Schmerz und Langeweile.

© 2000 Universität Leipzig (PD Dr. Jörg Schumacher & Prof. Dr. Elmar Brähler)

8. Wie oft sind Ihre Gedanken und Gefühle ganz durcheinander?

① ② ③ ④ ⑤ ⑥ ⑦

sehr oft — sehr selten oder nie

9. Kommt es vor, dass Sie Gefühle in sich haben, die Sie lieber nicht spüren würden?

① ② ③ ④ ⑤ ⑥ ⑦

sehr oft — sehr selten oder nie

10. Viele Leute - auch solche mit einem starken Charakter - fühlen sich in bestimmten Situationen als traurige Verlierer. Wie oft haben Sie sich in der Vergangenheit so gefühlt?

① ② ③ ④ ⑤ ⑥ ⑦

sehr oft — sehr selten oder nie

11. Wenn etwas passierte, hatten Sie dann im allgemeinen den Eindruck, dass Sie dessen Bedeutung ...

① ② ③ ④ ⑤ ⑥ ⑦

über- oder unterschätzten — richtig einschätzten

12. Wie oft haben Sie das Gefühl, dass die Dinge, die Sie im täglich Leben tun, wenig Sinn haben?

① ② ③ ④ ⑤ ⑥ ⑦

sehr oft — sehr selten oder nie

13. Wie häufig haben Sie Gefühle, bei denen Sie sich nicht sicher sind, ob Sie die unter Kontrolle halten können?

① ② ③ ④ ⑤ ⑥ ⑦

sehr oft — sehr selten oder nie

© 2000 Universität Leipzig (PD Dr. Jörg Schumacher & Prof. Dr. Elmar Brähler)

All rights reserved. For permission to use the scale please contact: Philip.moons@med.kuleuven.be

German translation: Corina Thomet, Susy Suter, Jacqueline Schwerzmann, Markus Schwerzmann, Kardiologie, Zentrum für angeborene Herzfehler, Inselspital, CH-3010 Bern/Schweiz. 2013

Gesundheitsverhalten

Dieser Fragebogen bezieht sich auf Ihr Gesundheitsverhalten. Bitte kreuzen Sie die richtige Antwort an. Bitte nur 1 Antwort pro Frage.

1. Konsumieren Sie Alkohol von Zeit zu Zeit? (unter Alkohol wird verstanden: Bier, Wein, Spirituosen, Alkopops, …)

- ☐ Nein (Weiter zu Frage 4)
- ☐ Ja

Falls ja, wie häufig?

- o Einmal im Monat oder weniger
- o 2 bis 4 mal im Monat
- o 2 bis 3 mal in Woche
- o 4 mal in der Woche oder häufiger

2. Wenn Sie Alkohol konsumieren, wieviel Gläser trinken Sie im Durchschnitt?

- o 1 bis 2
- o 3 bis 4
- o 5 bis 6
- o 7 bis 9
- o 10 oder mehr

3. Wie häufig trinken Sie 6 oder mehr Gläser bei einer Gelegenheit?

- o Nie
- o Seltener als einmal pro Monat
- o Monatlich
- o Wöchentlich
- o Täglich oder fast täglich

4. Rauchen Sie gelegentlich oder regelmässig Zigaretten?

- ☐ Nein (Weiter zu Frage 7)
- ☐ Ja

5. In den letzten 30 Tagen, an wieviel Tagen haben Sie Zigaretten geraucht?

- o An 1 bis 2 Tagen
- o An 3 bis 5 Tagen
- o An 6 bis 9 Tagen
- o An 10 bis 19 Tagen
- o An 20 bis 29 Tagen
- o An allen 30 Tagen

6. In den letzten 30 Tagen, an den Tagen an denen Sie geraucht haben, wieviel Zigaretten haben Sie pro Tag geraucht?

- o 1 Zigarette oder weniger pro Tag
- o 2 bis 5 Zigaretten pro Tag
- o 6 bis 10 Zigaretten pro Tag
- o 11 bis 20 Zigaretten pro Tag
- o Mehr als 20 Zigaretten pro Tag

7. Wie oft, in den vergangenen 12 Monaten, haben Sie folgende Drogen/Medikamente konsumiert?

	Nie	Einmal pro Monat oder weniger	2 bis 4 mal pro Monat	2 mal pro Woche oder häufiger
a. Cannabis (Marihuana, Haschisch)	○	○	○	○
b. XTC (Ecstasy)	○	○	○	○
c. Kokain	○	○	○	○
d. Halluzinogene Pilze	○	○	○	○
e. Speed	○	○	○	○
f. Schlaftabletten, Sedativa oder Beruhigungsmittel	○	○	○	○
g. Andere Drogen:	○	○	○	○

8. Waren Sie im vergangenen Jahr beim Zahnarzt?

☐ Nein

☐ Ja
(Weiter zu Fragen10 und 11)

9. Falls nein, wann waren Sie das letzte Mal beim Zahnarzt?

- o Ich gehe nie zum Zahnarzt
- o Vor 1-2 Jahren
- o Vor 2-3 Jahren
- o Vor mehr als 3 Jahren

10. Wie häufig putzen Sie sich Ihre Zähne?

- o Ich putze meine Zähne nicht
- o Ich putze meine Zähne hin und wieder
- o Einmal am Tag
- o Zweimal am Tag
- o Dreimal am Tag
- o Häufiger als dreimal am Tag

11. Wie häufig verwenden Sie Zahnseide?

- o Ich verwende keine Zahnseide
- o Ich verwende Zahnseide hin und wieder
- o Einmal pro Tag
- o Zweimal pro Tag
- o Dreimal pro Tag
- o Häufiger als dreimal pro Tag

12. Gehen Sie zu Fuss oder fahren Sie mit dem Velo regelmässig zur Schule oder an Ihren Arbeitsplatz?

☐ Nein
(Weiter zu Frage 14)

☐ Ja

13. Falls ja, wie lange benötigen Sie mit dem Velo oder zu Fuss (hin und zurück)?

- o < 15 min
- o 15-30 min
- o 30-45 min
- o > 45 min

14. Üben Sie regelmässig einen Sport aus (dies beinhaltet Schulsport, aber <u>NICHT</u> die Velo-oder Fussstrecke zur Schule oder zum Arbeitsplatz)

☐ Nein
(der Fragebogen endet hier, bitte gehen Sie weiter zur nächsten Seite)

☐ Ja

15. Während einer 7-Tage Woche, an wie viel Stunden üben Sie die folgenden körperlichen Aktivitäten aus?

a. <u>Sport in der Schule</u>, während dem Sportunterricht oder anderer Sport-Zeiten

Stunden/Woche ________________

b. Sportarten oder Aktivitäten, die körperlich <u>sehr anspruchsvoll</u> sind und Ihren Puls ansteigen lassen (z.B. Fussball, Langstreckenlauf, Basketball, Handball, Korbball, Squash, Rudern, Rugby, Hockey, Spinning, Thai Boxen, Kickboxen, Rennvelo fahren, Seilspringen, Mountainbiken, Tennis, ...)

Stunden/Woche ________________

c. Sportarten oder Aktivitäten, die körperlich <u>mässig anspruchsvoll</u> sind und Sie sich anschliessend nicht erschöpft und ausgebrannt fühlen (z.B. Joggen, Volleyball, Längen-Schwimmen, Ballett, Tanzen, Judo, Karate, Leichtathletik, Badminton, Baseball, Fitness-Stunden, Reiten, Sport-Klettern, …)

Stunden/Woche ________________

d. Sportarten oder Aktivitäten <u>mit minimaler körperlicher Anstrengung und leichten Belastungen</u> (z.B. Billiard, Kegeln oder Bowling, Darts, Golf, Kartenspiele, Yoga, Fischen, …)

Stunden/Woche ________________

ILLNESS PERCEPTION QUESTIONNAIRE (IPQ-R)
Kurzform

Name................................ *Datum....................................*

KRANKHEITSANNAHMEN

Mit diesem Fragebogen sollen Ihre persönlichen Annahmen und Vermutungen über Ihre Beschwerden erfragt werden. Bitte kreuzen Sie die zutreffenden Antworten an. Bitte beantworten Sie alle Fragen.

Im folgenden sind verschiedene Beschwerden aufgelistet, die Sie möglicherweise im Verlauf Ihrer Krankheit erlebt haben. Bitte geben Sie durch Ankreuzen an, ob Sie die betreffenden Symptome im Verlauf Ihrer Krankheit erlebt haben und ob Sie glauben, dass diese Symptome durch Ihre Krankheit verursacht werden.

	Ich habe dieses Symptom im Verlauf meiner Krankheit erlebt		Dieses Symptom wird durch meine Krankheit verursacht	
Schmerzen	Ja	Nein	Ja	Nein
Halsschmerzen	Ja	Nein	Ja	Nein
Übelkeit	Ja	Nein	Ja	Nein
Atemlosigkeit	Ja	Nein	Ja	Nein
Gewichtsverlust	Ja	Nein	Ja	Nein
Erschöpfung	Ja	Nein	Ja	Nein
Gelenkschmerzen	Ja	Nein	Ja	Nein
Brennende Augen	Ja	Nein	Ja	Nein
Kurzatmigkeit	Ja	Nein	Ja	Nein
Kopfschmerzen	Ja	Nein	Ja	Nein
Verdauungsprobleme	Ja	Nein	Ja	Nein
Schlafstörungen	Ja	Nein	Ja	Nein
Schwindel	Ja	Nein	Ja	Nein
Kraftlosigkeit	Ja	Nein	Ja	Nein

Wir sind daran interessiert, zu erfahren, wie Sie momentan Ihre aktuellen Krankheitssymptome einschätzen. Bitte geben Sie dazu durch Ankreuzen der betreffenden Antworten an, wie sehr Sie den folgenden Aussagen zustimmen oder diese ablehnen.

		STIMMT ÜBERHAUPT NICHT	STIMMT NICHT	WEDER NOCH	STIMMT	STIMMT VOLL UND GANZ
IP1	Meine Erkrankung ist eher chronisch als nur kurzzeitig					
IP2	Meine Krankheit wird lange Zeit andauern					
IP3	Ich nehme an, dass ich diese Krankheit für den Rest meines Lebens haben werde					
IP4	Mein Krankheit hat grosse Auswirkungen auf mein Leben					
IP5	Meine Krankheit hat grossen Einfluss darauf, wie andere Personen mich einschätzen					
IP6	Meine Krankheit verursacht auch Schwierigkeiten für mein soziales Umfeld					
IP7*	Nichts was ich tue, wird meine Krankheit beeinflussen					
IP8	Ich habe die Macht, meine Krankheit zu beeinflussen					
IP9*	Mein Verhalten wird den Verlauf meiner Krankheit nicht beeinflussen					
IP10*	Meine Krankheit ist für mich ein Rätsel					
IP11*	Ich verstehe meine Krankheit nicht					
IP12*	Meine Krankheit macht für mich keinen Sinn					
IP13	Meine Symptome kommen und gehen in einem wiederkehrenden Muster					
IP14	Meine Krankheit ist sehr unberechenbar					
IP15	Meine Krankheit hat einen phasenhaften Verlauf, bei dem es mal besser, mal schlechter ist					
IP16	Wenn ich über meine Krankheit nachdenke, fühle ich mich deprimiert					
IP17	Es beunruhigt mich, wenn ich über meine Krankheit nachdenke					
IP18	Es macht mich ängstlich, dass ich diese Krankheit habe					

KRANKHEITSURSACHEN

Bitte geben Sie uns zuletzt noch an, welche drei Faktoren Ihrer Meinung nach am meisten verantwortlich für Ihre Krankheit sind.

Die wichtigsten Ursachen meiner Krankheit sind:

1. ____
2. ____
3. ____

Hat sich Ihr allgemeines Befinden nach der Herzoperation im DHM verbessert?

- ☐ deutlich gebessert
- ☐ gering verbessert
- ☐ keine Änderung
- ☐ Verschlechterung des Befindens

Verspüren Sie zur Zeit Atemnot (NYHA)

- ☐ nach drei Stockwerken Treppensteigen
- ☐ nach einem Stockwerk Treppensteigen
- ☐ Gehen in der Ebene
- ☐ in Ruhe

Sind Sie verheiratet / in einer festen Partnerschaft? ☐ ja ☐ nein

Haben Sie leibliche Kinder? ☐ ja ☐ nein

Welchen Ausbildungsstand haben Sie?

- ☐ Studium
- ☐ Ausbildung
- ☐ keine Ausbildung nach Schulabschluss
- ☐ kein Schulabschluss

Sind Sie berufstätig?

- ☐ Ja, Vollzeit
- ☐ Ja, Teilzeit
- ☐ Nein, ich bin Hausfrau/Hausmann
- ☐ Nein, ich erhalte aktuell Krankengeld
- ☐ Nein, ich bin aktuell arbeitssuchend/arbeitslos
- ☐ Nein, ich bin in Rente

Haben Sie einen Führerschein? ☐ ja ☐ nein

Haben Sie folgende Versicherungen?

- ☐ Lebensversicherung
- ☐ Arbeitsunfähigkeitsversicherung

Beziehen Sie Leistungen aus der Pflegeversicherung?

- ☐ Nein
- ☐ Ja, ich habe Pflegestufe 0
- ☐ Ja, ich habe Pflegestufe 1
- ☐ Ja, ich habe Pflegestufe 2
- ☐ Ja, ich habe Pflegestufe 3
- ☐ Ja, aber ich kenne meine Pflegestufe nicht

Sonstiges

....
....
....
....
....

FU - Datum: .... **Unterschrift:** ....
