# Supplementary material for: Long-term patient-reported outcomes following congenital heart surgery in adults
Source: Front Cardiovasc Med. 2024 Dec 11;11:1501680. doi: 10.3389/fcvm.2024.1501680 (PMC11668799; doi:10.3389/fcvm.2024.1501680)
Supplement: Supplementary file 2 [file Table1.pdf]

**Supplementary Table 1**

**Patient-related outcomes for assessment of quality of life**

| Outcome | Questionnaire | Component | Scope | Min Score | Max Score |
|---|---|---|---|---|---|
| Perceived health status and Satisfaction of life | VR-12 | Mental Health | Emotions, vitality, mental and social functioning | 0 | 100 |
| | | Physical Health | Physical functioning, pain | 0 | 100 |
| | EQ-5D-5L | | Mobility, self-care, usual activities, pain, anxiety/depression | -0.661 | 1 |
| | | VAS | Perceived health status | 0 | 100 |
| | SWLS | | Life satisfaction, subjective well-being | 5 | 35 |
| Psychological functioning | PHQ-8 | | Depression | 0 | 24 |
| | SOC-13 | | Orientation to life (stress management, coping, resilience) | 13 | 91 |
| Health risk behaviors | HBS-CHD | Substance use | Alcohol drinking, smoking, drug abuse | 0 | 100 |
| | | Dental hygiene | Annual dental visit, daily brush, teeth floss | 0 | 100 |
| | | Overall risk | Substance use, dental hygiene, sport participation | 0 | 100 |
| Illness perception | IPQ-R | Consequences | Illness impact on one's life | 1 | 5 |
| | | Emotional representations | Negative emotions related to the illness | 1 | 5 |
| | | Identity | Attribution of symptoms specific to the illness | 0 | 28 |
| | | Coherence | Understanding of the illness (cause, treatment) | 1 | 5 |
| | | Personal Control | Self-efficacy to control symptoms | 1 | 5 |
| | | Timeline acute/chronic | Perceived duration of illness (short v/s long) | 1 | 5 |
| | | Timeline cyclical | Perceived duration of illness (cyclical) | 1 | 5 |

VR-12: Veterans RAND 12-item mental and physical scores, EQ-5D-5L: EuroQol 5-Dimension Level, SWLS: Satisfaction with Life Scale, PHQ-8: Patient Health Questionnaire, SOC-13: Sense of Coherence 13, HBS-CHD: Health-Behavior Scale–Congenital Heart Disease, IPQ-R: Illness Perception Questionnaire-Revised, VAS: visual analog scale. Blue and red values represent the best and worst scores, respectively, for each component of the questionnaires.
