# Supplementary material for: Long-term patient-reported outcomes following congenital heart surgery in adults
Source: Front Cardiovasc Med. 2024 Dec 11;11:1501680. doi: 10.3389/fcvm.2024.1501680 (PMC11668799; doi:10.3389/fcvm.2024.1501680)
Supplement: Supplementary file 3 [file Table2.pdf]

Supplementary Table 2

Primary Cardiac Diagnose	n (%)
ASD, secundum	34 (17.3)
TOF	22 (11.2)
Aortic insufficiency and aortic stenosis	11 (5.6)
Aortic stenosis, valvar	11 (5.6)
ASD, sinus venosus	10 (5.1)
Aortic aneurysm	9 (4.6)
Partial AVSD	8 (4.1)
Coarctation of aorta	6 (3.1)
Ebstein's anomaly	6 (3.1)
Pulmonary atresia, VSD	6 (3.1)
Mitral regurgitation	6 (3.1)
Aortic insufficiency	5 (2.6)
PAPVC	5 (2.6)
TGA, IVS	5 (2.6)
TGA, VSD-LVOTO	5 (2.6)
Pulmonary stenosis, valvar	4 (2.0)
Perimembranous VSD	4 (2.0)
TGA, VSD	3 (1.5)
DORV, TGA type	3 (1.5)
DORV, TOF type	3 (1.5)
DORV, VSD type	2 (1)
PFO	2 (1)
DCRV	2 (1)
Pulmonary atresia, IVS	2 (1)
Single ventricle, DILV	2 (1)
Single ventricle, tricuspid atresia	2 (1)
Transitional AVSD	1 (0.5)
Aortic stenosis, subvalvar	1 (0.5)
Congenitally corrected TGA	1 (0.5)
Anomalous aortic origin of coronary artery	1 (0.5)
Abnormal left coronary artery from pulmonary artery	1 (0.5)
DOLV	1 (0.5)
Double aortic arch	1 (0.5)
Interrupted aortic arch	1 (0.5)
Mitral stenosis, valvar	1 (0.5)
PAPVC, scimitar	1 (0.5)
Pulmonary insufficiency	1 (0.5)
Pulmonary stenosis, subvalvar	1 (0.5)
Pulmonary valve, other	1 (0.5)
Sinus of Valsalva aneurysm	1 (0.5)
TOF + AVSD	1 (0.5)
Truncus arteriosus	1 (0.5)
VSD + Coarctation of aorta	1 (0.5)
Infundibular VSD	1 (0.5)

ASD: atrium septum defect, TOF: tetralogy of Fallot, AVSD: atrioventricular septum defect, VSD: ventricular septum defect, PAPVC: partial anomaly of venous Connection, TGA: transposition of the great arteries, IVS: intact ventricular septum, LVOTO: left ventricular outflow tract obstruction, DORV: double-outlet right Ventricle, PFO: patent foramen ovale, DCRV: double-chambered right ventricle, DILV: double-inlet left ventricle, DOLV: double-outlet left ventricle
