# Supplementary material for: Long-term patient-reported outcomes following congenital heart surgery in adults
Source: Front Cardiovasc Med. 2024 Dec 11;11:1501680. doi: 10.3389/fcvm.2024.1501680 (PMC11668799; doi:10.3389/fcvm.2024.1501680)
Supplement: Supplementary file 4 [file Table3.pdf]

Supplementary Table 3

Cardiac Procedure in Adulthood	n (%)
ASD patch repair	31 (15.8)
RV to PA conduit placement	21 (10.7)
Mitral calvuloplasty	20 (10.2)
Conduit reoperation	12 (6.1)
PAPVC repair	12 (6.1)
Aortic valve replacement, mechanical	10 (5.1)
Aortic aneurysm repair	9 (4.6)
Tricuspid valvuloplasty	8 (4.1)
Aortic root replacement, valve sparing	7 (3.6)
Aortic root replacement, mechanical	6 (3.1)
Ebstein's repair	6 (3.1)
Aortic valve replacement, bioprosthetic	6 (3.1)
Fontan revision or conversion (Re-do Fontan)	4 (2)
Aortic valvuloplasty	4 (2)
ASD primary closure	3 (1.5)
Partial AVSD repair	3 (1.5)
Aortic root replacement, bioprosthetic	3 (1.5)
VSD patch repair	3 (1.5)
Tricuspid valve replacement	3 (1.5)
Atrial baffle procedure (Mustard or Senning revision)	2 (1)
Coarctation repair, interposition graft	2 (1)
PA reconstruction	2 (1)
RVOT procedure	2 (1)
Mitral valve replacement	2 (1)
Transitional AVSD repair	1 (0.5)
Left ventricular aneurysm repair	1 (0.5)
AAOCA repair	1 (0.5)
ALCAPA repair	1 (0.5)
Subvalvar aortic stenosis repair	1 (0.5)
Surgical ablation arrhythmia surgery (atrial)	1 (0.5)
DCRV repair	1 (0.5)
Konno procedure	1 (0.5)
PAPVC scimitar repair	1 (0.5)
Rastelli procedure	1 (0.5)
Systemic to pulmonary Shunt	1 (0.5)
Aneurysm of sinus of Valsalva repair	1 (0.5)
TOF repair without ventriculotomy	1 (0.5)
Pulmonic valve replacement	1 (0.5)
Truncal valve replacement	1 (0.5)

ASD: atrium septum defect, RV: right ventricle, PA: pulmonary Artery, PAPVC: partial anomaly of pulmonary venous connection, AVSD: atrioventricular septum defect, VSD: ventricle septum defect, RVOT: right ventricle outflow tract, AAOCA: anomalous aortic origin of the coronary artery from the

aorta, ALCAPA: anomalous origin of the coronary artery from the pulmonary artery, DCRV: double-chambered right ventricle, TOF: tetralogy of Fallot
